# Vitamin D deficiency as a potential risk factor for accelerated aging, impaired hippocampal neurogenesis and cognitive decline: a role for Wnt/β-catenin signaling

**DOI:** 10.18632/aging.103510

**Published:** 2020-06-17

**Authors:** Ricardo Gómez-Oliva, Noelia Geribaldi-Doldán, Samuel Domínguez-García, Livia Carrascal, Cristina Verástegui, Pedro Nunez-Abades, Carmen Castro

**Affiliations:** 1 Área de Fisiología, Facultad de Medicina, Universidad de Cádiz, Cádiz, Spain; 2 Instituto de Investigación e Innovación Biomédica de Cádiz, Cádiz, Spain; 3 Departamento de Anatomía y Embriología Humanas, Facultad de Medicina, Universidad de Cádiz, Cádiz, Spain; 4 Departamento de Fisiología, Facultad de Farmacia, Universidad de Sevilla, Sevilla, Spain

**Keywords:** vitamin D, neurogenesis, neural stem cells, cognitive performance, Wnt signaling

## Abstract

Vitamin D is an essential fat-soluble vitamin that participates in several homeostatic functions in mammalian organisms. Lower levels of vitamin D are produced in the older population, vitamin D deficiency being an accelerating factor for the progression of the aging process. In this review, we focus on the effect that vitamin D exerts in the aged brain paying special attention to the neurogenic process. Neurogenesis occurs in the adult brain in neurogenic regions, such as the dentate gyrus of the hippocampus (DG). This region generates new neurons that participate in cognitive tasks. The neurogenic rate in the DG is reduced in the aged brain because of a reduction in the number of neural stem cells (NSC). Homeostatic mechanisms controlled by the Wnt signaling pathway protect this pool of NSC from being depleted. We discuss in here the crosstalk between Wnt signaling and vitamin D, and hypothesize that hypovitaminosis might cause failure in the control of the neurogenic homeostatic mechanisms in the old brain leading to cognitive impairment. Understanding the relationship between vitamin D, neurogenesis and cognitive performance in the aged brain may facilitate prevention of cognitive decline and it can open a door into new therapeutic fields by perspectives in the elderly.

## INTRODUCTION

**Figure 1:**
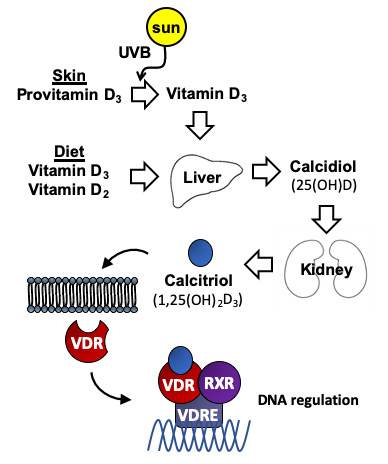
**Metabolism of vitamin D.** Vitamin D3 is synthesized in the skin from provitamin D3 (7-dehydrocholesterol) under the influence of UV light. Vitamin D2 (ergocalciferol) is obtained from vegetable dietary sources where it derives from the plant sterol ergosterol. Vitamin D is metabolized first to calcidiol (25(OH)D), and later to the active form calcitriol (1,25(OH)_2_D_3_). Interaction of 1,25(OH)_2_D_3_ with the vitamin D receptor (VDR), which is an intracellular transcription factor, facilitates its binding to DNA sequences. The binding of the complex VDR/1,25(OH)_2_D_3_ to these regulatory sequences (vitamin D response elements (VDREs)) regulate transcription of genes involved in many different cellular homeostatic functions.

Vitamin D is an essential fat-soluble vitamin that consists of two equally inactive forms, vitamin D_2_, also called ergocalciferol and vitamin D_3_ or cholecalciferol. Vitamin D2 can be obtained from vegetable dietary sources and food supplements, whereas vitamin D3 not only can be obtained from dietary sources, but it can also be produced by the human skin after exposure to ultraviolet B radiation in sunlight, with cutaneous production being the main source in the general population. As illustrated in Figure 1, either vitamin D_2_ or D_3_ must also be activated by transformations in the liver and kidney. Many variables influence the amount of ultraviolet B radiation from sunlight that reaches the skin and its effectiveness at facilitating the synthesis of vitamin D_3_. These variables include time of day, season, latitude, altitude, clothing, sunscreen use, pigmentation, and age. The activation of these vitamins into active metabolites occurs in two stages: the first stage is the hydroxylation of carbon 25 of vitamin D_2_ or D_3_ catalyzed by 25-hydroxylase leading to calcidiol (also called 25(OH)D or 25-hydroxyvitamin D) in the liver. The second stage is the transformation of 25(OH)D onto calcitriol (also called 1,25(OH)_2_D_3_ or 1,25-dihydroxyvitamin D), the most active form of vitamin D, catalyzed by 1α-hydroxylase mainly in the kidney. Although, classical function of vitamin D was always limited to calcium and phosphorus homeostasis, the discovery of vitamin D receptor (VDR), present in most tissues and cells in the body, including the brain [1, 2], meant an increase in the number of studies focusing on vitamin D functions. VDR can regulate a large number of genes through 1,25(OH)_2_D_3_. The binding of 1,25(OH)_2_D_3_ to VDR generates a cytosolic complex that regulates gene transcription and many biological functions (Figure 1). VDR/1,25(OH)2D_3_ complex can interact with retinoid X receptor (RXR) in the cytosol to form a heterodimeric complex which is recruited to the VDRE (Vitamin D Receptor Element) placed in the promoters of target genes to activate its regulation [3] (Figure 1). The best well known function of 1,25(OH)_2_D_3_ is the regulation of calcium homeostasis and bone mineralization. However, ontology analysis describe 11,031 putative VDR target genes identified, 43% of which were involved with metabolism, 19% with cell and tissue morphology, 10% with cell junction and adhesion, 10% with differentiation and development, 9% with angiogenesis, and 5% with epithelial to mesenchymal transition [4]. The number, the location and the VDR expression regulation are determined by cell type [5–7]. These genes are involved in several processes such as cell proliferation, cancer, immune response, glucose homeostasis, cardiovascular homeostasis and activity of the nervous system [8–11].

In the human brain, VDR and vitamin D-metabolizing enzymes are expressed by cerebral structures such as prefrontal cortex, hippocampus, cingulate gyrus, thalamus, hypothalamus, and substantia nigra [12, 13]. In neurons, vitamin D plays different key roles participating in the suppression of oxidative stress, inhibition of inflammation, neuroprotection, down-regulating inflammatory mediators and up-regulating many neurotrophins [13, 14]. Proteomics and gene array analyses show that low levels of vitamin D during gestation influence the regulation of genes involved in nervous system development. These genes play significant roles in the cytoskeletal maintenance, mitochondrial function, synaptic plasticity and cellular proliferation and growth [15]. Regarding neurotrophins, vitamin D exerts neurotrophic support participating in the synthesis of neurotrophic factors. It participates in the synthesis of nerve growth factor (NGF) upregulates, the synthesis of glial cell line-derived neurotrophic factor (GDNF) and neurotrophin 3 (NT-3), and also downregulates levels of neurotrophin 4 (NT-4) [9, 16, 17].

Although there are probably around 50 known metabolites of vitamin D, measurement of serum 25(OH)D is clinically used to define the vitamin D status. The threshold to define adequate stores of 25(OH)D in humans has not been clearly established. Diversity of opinions among researchers has generated different thresholds of vitamin D for human health. Thus, the Institute of Medicine has stablished the optimal concentration of 25(OH)D serum level being 50 nM (20 ng/mL) for skeletal health [18], insufficiency between 30–50 nM, and deficiency below 30 nM (12 ng/ml), whereas the International Osteoporosis Foundation considers that the adequate values of 25(OH)D for skeletal health are higher than 75 nM (30 ng/mL) [19]. Despite the lack of consensus, it is clear that low levels of vitamin D have detrimental consequences for human health [20].

Though the main source of vitamin D is the sunlight, its deficiency has a high prevalence worldwide and affects half of the world population without excluding those in countries with sun exposure over all the year [21, 22] thus leading to a great variety of health problems. In addition to the widely studied actions of 1,25(OH)_2_D_3_ on intestinal calcium absorption and bone physiology, studies in animal models show that 1,25(OH)_2_D_3_ exerts tumor-suppressive actions (anti-angiogenic, anti-invasive, antimetastatic) [23] in several cancers and epidemiological studies report that vitamin D exerts protective effects against several neoplasia, particularly colorectal cancer [24]. Vitamin D deficiency is associated as well with several brain diseases such as schizophrenia, autism spectrum disorders, multiple sclerosis, dementia and Alzheimer’s disease [25–33].

## Vitamin D deficiency and accelerated aging

Several studies start to consider vitamin D deficiency as a risk factor for accelerated aging [11, 34–36] especially in the elderly [37] since the body reduces its ability to synthesize 1,25(OH)_2_D_3_. The skin’s ability to synthesize vitamin D significantly decreases with age, being reduced by more than 50% at 70 years of age compared to 20, whereas other functions such as the intestinal absorption of vitamin D are not affected [38]. Moreover, several studies have reported that hypovitaminosis D is common in aged individuals with previous diseases [39].

Aging is considered to be controlled by multiple genes and environmental factors, and vitamin D is postulated as one of these key factors. Keisala et al. show a direct connection between VDR and aging demonstrating that the phenotype of VDR KO mice includes premature aging and a shorter life span [40]. In addition, VDR KO mice manifest some of the health problems observed during the human aging process such as infertility, muscle atrophy, immune deficiency, osteoporosis and sensitivity to cancer [10, 41, 42]. Additional studies reveal that in addition to a shorter lifespan, VDR mutant mice show other signs of accelerated aging such as skin thickening and wrinkling, alopecia, ectopic calcification, progressive loss of hearing and balance [43, 44]. It is proposed that vitamin D regulates aging by controlling several cell activities such as autophagy, which acts to slow down the aging process by removing dysfunctional mitochondria. Vitamin D also moderates oxidative stress, inflammation, calcium signaling, epigenetics and DNA disorders, including telomere shortening that leads the processes of aging [11, 45–49].

All of this suggests that vitamin D is essential for the maintenance of homeostasis during aging and its deficiency might accelerate its progression. These evidences together with the reduced capacity of human skin to produce vitamin D_3_ during aging allow to propose a feedback positive loop between vitamin D deficiency and aging: aging provokes more vitamin D deficiency and vitamin D deficiency accelerates the aging process. In the next paragraphs of this review, we will focus on the effect that vitamin D exerts in the aged brain paying special attention to the neurogenic process.

## Hippocampal neurogenesis in the aged brain

Neurogenesis occurs during development of the central nervous system and remains during the infant and adult stages. New neurons are generated from neural stem cells (NSC) which produce glial cells as well. NSC are ubiquitously distributed along the adult central nervous system [50], however, once the brain has completely developed, neurogenesis predominantly occurs in two specific regions of the adult mammalian brain: the subventricular zone (SVZ) and the dentate gyrus of hippocampus (DG) [51, 52]; nonetheless, there are other minor scattered sites in the brain where neurogenesis occurs such as the hypothalamus or the striatum of several species [53–55]. Within these regions an environment of extracellular signaling molecules creates a neurogenic niche that preserves the necessary conditions to support neurogenesis during a lifetime. Different cell types derived from the NSC progeny can be distinguished within these niches: undifferentiated neural progenitor cells (NPC) produced by activated NSC, and neuronal progenitor cells (neuroblasts) that differentiate into mature neurons. Since the potentiality of NPC is almost identical to that of NSC, they can produce either neuronal progenitors or glial progenitors [56–58] and the fate of NSC may determine the neurogenic capacity of the hippocampus in the long term. NSC activated in the DG undergo a series of asymmetric divisions that produce neurons until they eventually differentiate into astrocytes [59]; thus, the proportion of glial cells produced from NSC over neurons in the DG varies with age and a biased differentiation of NSC towards an astroglial phenotype has been shown in the DG of aged mice, which leads to a depletion of the NSC pool and a reduction of neurogenesis [59, 60]. Extracellular, matrix-bound and membrane-bound signals determine NSC fate toward a neuronal or glial phenotype within the niche [61]. One of this signals is the brain morphogenetic protein (BMP) signaling inhibitor Noggin, which is a key molecule protecting NSC in the aged brain because of its role in the regulation of BMP signaling [60]. Other signaling molecules involved in fate determination are those that initiate the epidermal growth factor receptor (EGFR) or the basic fibroblast growth factor (bFGF) pathways [61–65], which might be stimulated by intracellular signaling molecules such as classical and novel protein kinase C isozymes [66, 67].

Since the year 1965 in which a study about the generation of neurons in the postnatal mammalian brain was reported, neurogenesis in the adult has been a controversial point [68]. The persistence of hippocampal neurogenesis in the adult mammalian brain has been demonstrated in rodents and other mammalian species, however, a dramatic decline of the rate at which new neurons are generated in older animals has also been observed [51, 69, 70]. Proliferating cells in the subgranular zone (SGZ) of the hippocampus rapidly decline in early childhood [71, 72]. Moreover, the amount of gliogenesis increases whereas that of neurogenesis decreases during aging [70, 73]. Nowadays, the debate continues determining whether human hippocampal neurogenesis remains active during physiological aging. The greatest culmination of this debate has recently come with two very contradictory studies [74, 75]. Both studies were based in the same premise and used a variety of similar antibodies to detect markers of NSC, proliferating cells, migrating neural cells, and various stages of neuronal maturation. One of them concludes that there are undetectable levels of hippocampal neurogenesis in adult brains [74] whereas two other studies conclude that human hippocampal neurogenesis persists throughout adulthood [76] and even in aged adults [75]. Today, the argument that adult neurogenesis persists in the human hippocampus has more adepts, based on BrdU marker and carbon dating but a deeper study on hippocampal adult neurogenesis should be done. Ideally, non-invasive *in vivo* techniques could be used to detect neurogenesis such as magnetic resonance imaging and positron emission tomography in living humans. This field is under development and there are already some studies describing *in vivo* imaging of endogenous NPC using these techniques [77].

A key point in the regulation of neurogenesis within neurogenic niches is whether NSC adopt a quiescent state or enter an active state. NSC are exposed to a large variety of signals from the environment, either inhibitory or stimulating, which they integrate resulting in either the maintenance of the quiescent state (qNSC) or the transition into an activated state (aNSC) [78]. These are extracellular matrix, cell-bound or soluble paracrine signals. Interestingly some of these signals regulate stem cells in different tissues in a similar manner; i.e. BMPs promote quiescence whereas activation of the Wnt signaling pathway promotes activity of various types of stem cells [78].

Recent studies demonstrate that the number of hippocampal NSC decreases with age and concomitantly, these cells undergo a transition into a senescent state characterized by a complex morphology. The capacity of these senescent cells to undergo activation is greatly reduced. Thus, NSC remain quiescent for longer periods of time in the DG of aged adults [79, 80]. The quiescence maintenance is probably the major factor contributing to the preservation of the neurogenic rate during aging since it protects NSC reservoir from full depletion. However, a basal activation rate is required for the continuous generation of new neurons. Within this context, the niche plays a major role in reducing qNSC activation in the aged brain. Recent works have demonstrated that inflammatory signals within the aged DG niche may increase quiescence in NSC [79] and have elucidated some of the cellular and molecular mechanisms underlying this phenomenon [80], which include the hypomethylation of genes involved in the Wnt signaling pathway stabilizing the expression profile of some of its components [80].

In general, most studies yield to the aging-induced quiescence conclusion and therefore it seems reasonable to hypothesize that in order to control NSC aging, it is important to regulate the balance between quiescence and activation of NSC by understanding the role of the signals within the niches that can lead NSC to exit quiescence [81]. Interestingly, in addition to the inflammatory signals and cascades that modulate NSC quiescence and activation, the Wnt signaling pathway seems to play a crucial role in regulating the balance quiescence/activation. Several Wnt signaling proteins participate in this process [78, 80].

## Neurogenesis and Wnt signaling

### Wnt signaling pathways in the central nervous system

Typically, Wnt proteins play essential roles in different signaling pathways in cellular proliferation, differentiation and cell migration during central nervous system development but recently, studies have shown that Wnt signaling is not only implicated in embryonic development but also in the adult state. Wnt ligands are constitutively expressed in the adult brain and have a role at least in the maintenance of adult brain neurogenesis [82, 83]. The active role of Wnt during brain development is regulating neurogenesis and synaptogenesis of the neural tube [84]. Therefore, constitutive expression of Wnt ligands in the hippocampus of the adult brain might suggest an important role for Wnt in the maintenance and protection of adult hippocampal neurogenesis during adulthood. Moreover, it has been suggested that its deregulation is crucial in neurogenesis during aging [85] and in several neurological disorders such as Alzheimer’s disease, Parkinson’s disease or Schizophrenia [86]

The importance of Wnt signaling can be inferred from the conservation of this pathway in the different organisms across evolution including humans. There are three Wnt stimulated pathways very well characterized: the canonical β-catenin dependent Wnt pathway, the noncanonical β-catenin independent pathways: planar cell polarity pathway (PCP) and calcium pathway (CaP). These pathways are activated by the binding of a Wnt ligand (Wnt 1-19) to a Frizzled (Fzd) receptor and the LDL-Receptor-related protein coreceptor (LRP5/6) resulting in the activation of Disheveled (DVL) protein which initiate different signaling cascades [87–89].

**Figure 2:**
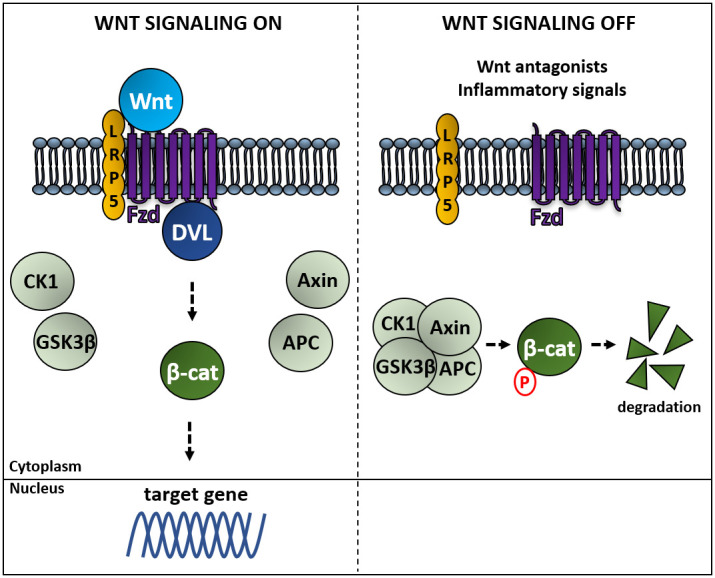
**Activation of the Wnt canonical pathway induces β-catenin-regulated gene expression.** Left panel: binding of Wnt to a Frizzled receptor (Fzd) allows its association to Dishevelled proteins (DVL) sequestering Axin and avoiding the formation of the complex composed of Axin, the adenomatous polyposis coli (APC), the kinases casein kinase 1 (CK1) and glycogen synthase kinase 3 beta (GSK3β), which phosphorylates β-catenin, resulting in β-catenin being ubiquitinated by the β-Trcp ubiquitin ligase, followed by proteasomal degradation. Right panel: in the absence of Wnt β-catenin is degraded, whereas Wnt-mediated activation of Fzd induces expression of genes regulated by β-catenin [92].

In the Wnt canonical pathway, Fzd and DVL activation avoid the degradation of β-catenin (Figure 2). In the absence of Wnt, β-catenin is continuously degraded by a protein complex composed of the scaffold proteins Axin and adenomatous polyposis coli (APC), and the kinases casein kinase 1 (CK1) and glycogen synthase kinase 3 beta (GSK3β). CK1 and GSK3β sequentially phosphorylate β-catenin, resulting in β-catenin being recognized and ubiquitinated by the β-Trcp ubiquitin ligase, followed by proteasomal degradation. Binding of Wnt to a Fzd receptor complex induces the binding of Dvl to Fzd and the recruitment of Axin to the membrane, which impairs the destruction complex through the inactivation of GSK3β, promotes the release of β-catenin, and its accumulation in cytoplasm and nuclei leading to β-catenin-activated gene expression [90–92]. Inhibitors of this pathway are the Dickkopf proteins 1-4 (DKK 1-4) and the secreted frizzled related proteins 1-5 (SFRP 1-5) [88]. This branch of the Wnt signaling pathway plays key roles in regulating cell fate, proliferation and survival [93]. The noncanonical pathways PCP and CaP are activated by Wnt4, Wnt5a and Wnt11. These pathways control gene expression through different mechanisms involving RhoA/Rock kinases or the calmodulin kinase CamKII respectively [94]. This branch is more associated with differentiation, cell polarity and migration [93]

### Wnt and adult neurogenesis

In multiple mammalian tissues canonical Wnt signals within the niche act as self-renewal short range signals for stem cells tissues [89]. There is also increased evidence about Wnt involvement in adult neurogenesis. It has been demonstrated that adult hippocampal progenitor cells express different Wnt ligands which can regulate adult hippocampal neurogenesis acting on both canonical and non-canonical signaling pathways. Recent studies indicate that two major branches of the Wnt signaling pathway, the Wnt/β-Catenin and Wnt/PCP pathways, play essential roles in various steps of adult neurogenesis [86]. However, at least 19 Wnt proteins and 10 Fzd receptors have been found [95]. This diversity of signals and receptors complicate the comprehension of the impact of their different roles in mammals.

The overexpression of Wnt ligands that activate the canonical Wnt β-catenin pathway such as Wnt3 increases neurogenesis of adult hippocampal progenitor cells *in vitro* and *in vivo*, suggesting that Wnt signaling enhances proliferation of neural stem cells derived from adult CNS [96, 97]. In agreement with this, Wnt3 signaling inhibition blocks neurogenesis in the DG and decreases long-term retention of episodic memory in adult rats [98]. Accordingly, the deletion of Wnt7a reduced drastically the numbers of newborn neurons in the DG of adult mouse brains preventing NPC proliferation and differentiation through the canonical Wnt/β-catenin pathway [99]. In addition, the implication of Wnt pathway is not only revealed by Wnt ligands but also by their Fzd receptors. Fzd1 knockdown reduces the generation of newborn neurons in the DG and changes the migration of neurons [100]. Another Wnt canonical signaling regulator that participates in adult neurogenesis is GSK3β. Overexpression of this kinase inhibits neurogenesis in the adult DG whereas its inhibition facilitates NSC proliferation and neuronal differentiation (reviewed in Marchetti et al. 2020 [86]). Finally, Wnt-signaling must be finely tuned via Wnt-antagonists such as some Dikkopf (DKK) proteins. Dkk1 is a potent inhibitor of SVZ- and SGZ-neurogenesis [83].

An additional role for non-canonical Wnt signaling pathway has also been reported. Wnt5a knockdown in the mouse DG impaired neuronal differentiation of progenitor cells and reduced dendritic development of adult-born neurons. In cultured adult hippocampal progenitors, knockdown of noncanonical Wnt5a reduced neuronal differentiation and morphological development of adult neurons, whereas treatment with Wnt5a had the opposite effect. Arredondo et al. determined that Wnt5a signals through CaMKII induce neurogenesis and promotes dendritic development of newborn neurons through activating Wnt/JNK and Wnt/CaMKII signaling suggesting that Wnt5a act as a niche factor in the adult hippocampus that promotes neuronal differentiation and development [101].

Altogether these evidences support the relevance of Wnt signaling pathway on adult neurogenesis. However, the understanding of the complex regulation of Wnt signaling in neurogenesis in the adult brain remains unclear.

### Wnt and the aged brain

It has been proposed that neurogenesis could be finely regulated by the expression of specific Wnt receptors in different cell types in young adults and this regulation is altered in the aged brain [102]. In the young adult, hippocampal astrocytes express Wnt3, which stimulates the canonical β-catenin pathway in neuroblasts promoting proliferation and differentiation via paracrine signaling [97]. Autocrine Wnt signals in NSC and NPC within the DG maintain their proliferative activity [103, 104] via the β-catenin pathway. Furthermore, mature granule neurons in the DG express the Wnt inhibitor sFRP3. The expression of this inhibitor can be greatly reduced depending on neuronal activity leading to proliferation of NPC and maturation of newly generate neurons [105]. Non canonical PCP Wnt signaling also plays an important role in neurogenesis in the young adult by inducing neuroblast differentiation and migration [106]. Wnt activity is different in the aged DG compared to the young adult. A reduction in canonical Wnt activity has been described in the hippocampus of aged animals. Wnt3 expression of hippocampal astrocytes and the number of Wnt3-secreting astrocytes is reduced during aging [107]. Decreased Wnt levels together with an elevated expression of Wnt antagonists, such as DKK1, could partially explain the decline in neurogenesis found in aged adults [107–109]. Loss of the Wnt antagonist DKK1 in aged KO mice results in a restoration of the decline in neurogenesis found in non-mutant aged mice. [109]

An attenuation of Wnt signaling has also been found in the SVZ. Zhu et al. detected decreased canonical Wnt activity in the SVZ of old mice compared to younger mice that could be responsible for the reduced adult neurogenesis in rodents [110]. A negative regulator of Wnt is the p38 mitogen-activated kinase (p38 MAPK), which inactivates GSK3β leading to the attenuation of Wnt signaling. Kase et al. have identified p38 MAPK as a key factor in the proliferation of NPC in adult neurogenic niches. p38 expression in adult NSC/NPC is downregulated during aging. Deletion of p38α in NSC/NPC specifically reduces the proliferation of NPC but not stem cells. Overexpression of p38α in NSC/NPC in the aged mouse SVZ restores NPC proliferation and neurogenesis and prevents age-dependent SVZ atrophy [111].

An effect of Wnt on the transition from qNSC to aNSC that is altered in the aged brain has also been proposed. However, this subject is still an open question. Some evidences point out at a role for canonical Wnt signaling in promoting activation of NSC in the SVZ and DG. Wnt signals produced by astrocytes and NSC induce proliferation and self-renewal of NSC in both niches [97, 99]. Also, elimination of sfrp3 expressed in hippocampal granule neurons results in aberrant NSC activation. Accordingly, sfrp3 gradients regulate qNSC activation regionally. A similar effect is observed upon elimination of DKK1, a Wnt inhibitor expressed by NPC within the hippocampus [105, 109, 112]. In addition, some studies suggest that non-canonical Wnt signaling maintains quiescence of SVZ NSC by facilitating anchoring of NSC within the niche in a mechanism mediated by Rho GTPase Cdc42 [113]. All these suggests that activation requires a switch from non-canonical to canonical Wnt signaling [78]. Wnt signaling molecules have been found to be altered in the pathogenesis of aging. In fact, p38-MAPK is necessary for suppressing the expression of sfrp3 and other Wnt antagonists like DKK1, which inhibit the proliferation of NPCs, and therefore, an age-related reduction in p38 leads to decreased adult neurogenesis via downregulation of Wnt signaling [111].

Studies using mathematical models show that in mice in which the Wnt antagonist DKK1 has been deleted, NSC spend longer periods of time in quiescence but they are more likely to be activated than depleted via their differentiation towards astroglial cells [114]. The study concludes that, high NSC-Wnt activity leads to longer time in quiescence while enhancing the probability of activation.

## Crosstalk between Wnt signaling and vitamin D

**Figure 3:**
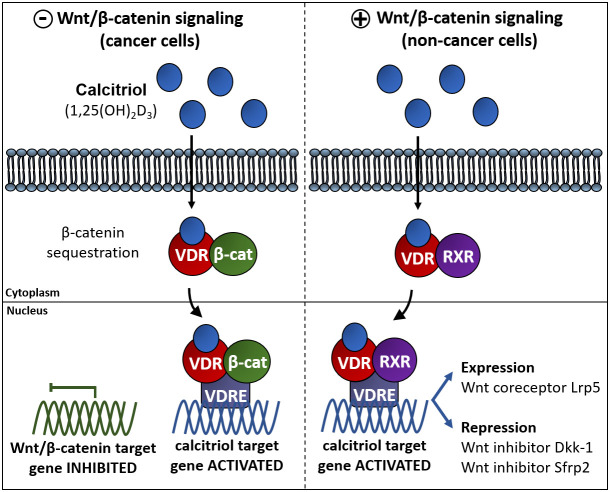
**Vitamin D interferes with β-catenin induced gene expression via different pathways in different cell types.** Left panel: in cancer cells vitamin D impairs the Wnt/β-catenin signaling pathway. One of these mechanisms relays on the association of the complex VDR/1,25(OH)_2_D_3_ to β-catenin to induce VDR-regulated gene expression avoiding β-catenin dependent gene expression. Right panel: in some other non-cancer cell types vitamin D exerts an activating effect of the Wnt signaling pathway by upregulating the expression of the Fzd co-activator Lrp5 or by repressing the expression of the Wnt inhibitors DKK1 y Sfrp2 [116, 171].

The activation of VDR depends on the presence of 1,25(OH)_2_D_3_ which triggers the direct regulation of genes with VDRE (as illustrated in Figure 3). But in some cases, 1,25(OH)_2_D_3_ can also indirectly regulate genes that do not contain VDRE in their promoters because 1,25(OH)_2_D_3_/VDR can also regulate other pathways through β-catenin which is required for gene expression in response to Wnt signaling (Figure 3). The relationship of this crosstalk is complex and not fully understood in all tissues and cells. The crosstalk between 1,25(OH)_2_D_3_ and Wnt/β-catenin pathway has been reported in cancer cells, for example *in vitro* functional validation studies on melanoma and colon cancer cells showed that elevated 1,25(OH)_2_D_3_/VDR signaling inhibit Wnt/β-catenin signaling genes [24, 115, 116]. Besides, interactions between vitamin D and Wnt/β-catenin pathway has also been reported in different cellular contexts such as colon cancer cells [117], in which 1,25(OH)_2_D_3_ acts upregulating the extracellular Wnt inhibitor DKK1 antagonizing of Wnt/β-catenin pathway [118], promoting VDR/β-catenin interactions [119], thus reducing the β-catenin-dependent gene expression or facilitating the sequestration of β-catenin by E-cadherin at plasma membrane adherents junction [117, 119, 120]. Similar mechanisms have been described in other cell types [121, 122].

The 1,25(OH)_2_D_3_-induced repression of β-catenin is not the only mechanism of action of 1,25(OH)_2_D_3_ in the Wnt signaling pathway (Figure 3, left). Interestingly, an upregulation of the Wnt/β-catenin pathway by VDR has been described in osteoblasts and keratinocytes in which 1,25(OH)_2_D_3_ effects are similar to those of Wnt: 1,25(OH)_2_D_3_ induces the expression of the Wnt coreceptor Lrp5 in mouse osteoblast [123] while represses Wnt inhibitors Dkk-1 and Sfrp2 in mesenchymal stem cells (Figure 3, right). In skin, deficiency of VDR produces hair loss and a gradual decrease in epidermal stem cells, while the transcriptional effects of β-catenin are impaired thus normal postnatal hair cycling is only possible with combined action of these two pathways [124]. VDR acts as a Wnt effector and β-catenin as a co-activator to induce transcription of genes involved in the hair follicle differentiation [125]. Theses evidences show that Wnt/β-catenin and 1,25(OH)_2_D_3_ can work together or are linked to regulate their target genes. The mechanism of action diverges depending on the biological system. While vitamin D can act as an antagonist of the Wnt/β-catenin pathway in some cancer cells, it can also act as co-activator of Wnt/β-catenin pathway in other physiological cell types.

Considering the cross talk between vitamin D and Wnt pathway and the considerable number of reports demonstrating a role for Wnt signaling in the regulation of neurogenesis, it would be reasonable to hypothesize that vitamin D may play a role in adult neurogenesis affecting brain tasks associated with neurogenesis such as cognitive performance.

## Axis vitamin D deficiency, cognitive decline and neurogenesis in the aged brain

Nowadays, it is well established that hippocampal neurogenesis is involved in learning and memory; studies where hippocampal neurogenesis was ablated in rodents have shown diminished performance in tests that require memory such as the Morris water maze, spatial and object recognition and pattern separation [99, 126, 127]. Furthermore, adult hippocampal neurogenesis has been linked to cognitive abilities both in rodents and in non-human primates [128]. In the human hippocampus, neurogenesis is still a controversial subject. Recent study suggests that hippocampal neurogenesis declines at young ages to disappear in the adult [74] and a similar decline has also been observed in the hippocampus of other large brain species [129]. However, several evidences show that new neurons can be generated daily throughout the lifespan [75, 76] suggesting a possible functional role for hippocampal neurogenesis in human cognitive capacity [70, 74]. Some studies define potential cognitive functions of new neurons of the hippocampal formation including the ability to discriminate among similar experiences. In fact, neurogenesis functions in fear conditioning are especially striking in discriminative paradigms, where shock is associated with only one of two similar-appearing situations. Consistent with a discrimination function, adult mice where hippocampal neurogenesis is ablated or deficient are frequently capable of initial learning in spatial tasks but have difficulty performing a spatial reversal or discriminating nearby locations or cues [126]. Another potential cognitive function of hippocampal neurons is incorporating time into episodic memories and enabling forgetting of old memories. Increasing neurogenesis after the formation of a memory was enough to induce forgetting in adult mice. Accordingly, during infancy, when hippocampal neurogenesis levels are high and freshly generated, memories tend to be rapidly forgotten (infantile amnesia), decreasing neurogenesis after memory formation mitigated forgetting [130]. Hippocampus-dependent cognitive abilities decline with age in human at the same time that adult hippocampal neurogenesis [51, 131, 132]. Most of these studies use rodent models and they suggest a similar scenario may occur in humans although recent data suggest that maybe age-related decline is not so pronounced in humans [70]. Furthermore, cognitive functions can be regulated by certain positive and negative modulators of hippocampal neurogenesis. Inflammatory signals negatively affect neurogenesis and therefore, considering that chronic neuroinflammation is a common feature of normal aging, hippocampal neurogenesis and cognitive processes would be negatively affected across the lifespan [133–135]. On the contrary exercise training and environmental enrichment have been suggested as positive factors since it has been demonstrated that both situations stimulate hippocampal neurogenesis [136–138] and improve cognitive function [139–141]. Besides, recent evidences demonstrated that exercise program can be intergenerationally inherited. Among others, these inherited effects include: improving the performance of non-spatial and spatial cognitive tasks, increasing the number of specific cell populations of adult hippocampal neurogenesis and producing changes in hippocampal gene expression [142].

Vitamin D deficiency is a risk factor for accelerated aging and cognitive decline [11, 34–36]. In addition, several studies suggest that low levels of vitamin D are associated with a substantial cognitive decline in the elderly [143, 144]. Aging processes in vitamin D deficient subjects could also promote the beginning of many age-related disorders such as a decline in cognition, depression, osteoporosis, hypertension and cardiovascular disease, diabetes, cancer, muscle weakness, and Alzheimer’s disease [145–151]. In light of these findings some authors propose to use 25(OH)D sufficiency as a biomarker of delayed aging [152] and some others propose vitamin D supplementation as a possible therapeutic agent for the treatment of age-related disorders such as cognitive decline [153]. Recent findings suggest vitamin D deficiency as a risk for cognitive decline in elderly people. Low vitamin D levels (<25 nmol/L) have been associated with a cognitive decline in aged individuals studied over a 6-year period [154]. Similarly, in a different study, low 25(OH)D levels (<35 nmol/L) were associated with poorer performance on cognitive test in older European men [155]. In addition, other studies show that 25(OH)D (<50 nmol/L) is strongly associated with executive functioning and the attention processing speed but no association between 25(OH)D and memory were found [156]. Whereas another clinical study with 1604 elderly men found no significant association between low vitamin D levels (<50 nmol/L) and cognitive decline after adjusting for co-variates [157]. In agreement with this latter study, Lee et al. did not find a direct correlation between vitamin D deficiency and cognitive impairment although they did not discard that vitamin D could be an important covariable factor [158].

Thus, the emerging evidences that suggest associations between lower serum vitamin D concentrations and poor cognitive performance have recently increased. Occasionally, vitamin D levels could be normal but insufficient to accomplish its function. This is the case of impaired VDR function. It is known that VDR gene polymorphisms decrease the VDR affinity for vitamin D but in contrast to vitamin D deficiency studies, little is known about the influence of VDR genes on cognition. Evidences point to the VDR gene variants being linked to changes in cognitive performance in old adults [159, 160]. A clinical study with 563 85-year-old participants showed cognitive differences depending on polymorphisms in the VDR gene [161]. VDR polymorphisms influence susceptibility for cognitive decline in average, 67.4 years old patients with Parkinson’s disease. Particularly, the functional VDR polymorphism Fokl, is associated with cognitive decline in patients with Parkinson’s disease, which worsen with each additional copy of the allele [162]. VDR is expressed in human brain [163] covering a large area including the hippocampus [164] which is partially involved in cognitive abilities and is particularly affected by neurodegenerative disorders.

Besides the link between vitamin D effectivity (vitamin D deficiency or VDR polymorphism) and cognitive decline the mechanism underlying is poorly understood. 1,25(OH)_2_D_3_ exerts a direct effect on NSC proliferation, survival, and neuron/oligodendrocyte differentiation participating in the process of remyelination [31–33]. Other studies using different models of knockout mice show an effect of vitamin D deficiency on adult hippocampal neurogenesis. 1α-hydroxylase knockout mice, which lacks the ability to produce the active form of vitamin D (1,25(OH)_2_D_3_) [165] and BALB/c mice fed a vitamin D deficient diet [166] show an increase in neuroblast proliferation in the hippocampal DG, but a decrease in the survival of adult hippocampal neurons. Moreover, it has also been observed alterations in neuronal differentiation not only in VDR deficient mice [167] but also in a mice model of Parkinson’s disease in which MPTP downregulates VDR expression [168]. An effect of vitamin D in neuronal differentiation of dopamine systems during development has been described [169]. Accordingly, nutritional supplementation with vitamin D in a mouse model of Alzheimer’s disease improves cognition concomitantly enhancing neurogenesis [170]. Also, it facilitates differentiation and neurite outgrowth of HN9.10e embryonic hippocampal cells [168].

Altogether these findings suggest a role for vitamin D in preserving cognitive function in older adults and indicate that vitamin D is not only related with aging but also with cognitive performance. Hence, it seems reasonable to hypothesize that the cellular mechanisms underlying the effects of vitamin D on cognitive performance in the elderly might be mediated by its capacity to stimulate neurogenesis. They also highlight a role for canonical Wnt signaling cascade as the molecular mechanisms triggering these effects. Considering the role that canonical Wnt signaling plays in stimulating neurogenesis in the aged brain and in maintaining the balance between aNSC/qNSC avoiding depletion, it may be possible that a deficiency in vitamin D results in Wnt signaling imbalance, impairing the gradual activation of NSC required to maintain a neurogenic rate. However, more studies are required to demonstrate this hypothesis. Only a few human trials have been performed to analyze the benefits of vitamin D supplementation in cognitive performance. However, evidences suggest a beneficial role for vitamin D in brain physiology by the promotion of neurotransmission, neurogenesis, synaptogenesis, amyloid clearance and the prevention of neuronal death [153].

## CONCLUSIONS

**Figure 4:**
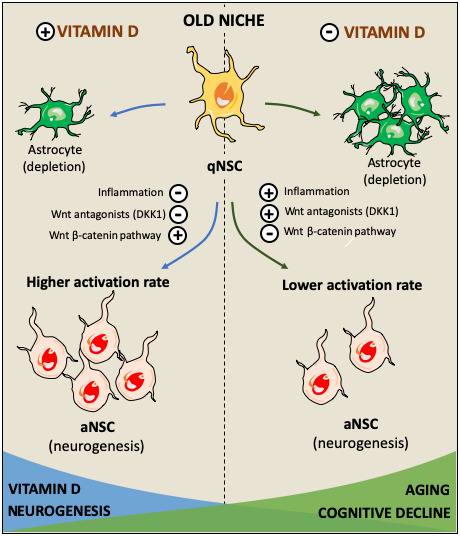
**Hypothetical role of vitamin D in facilitating the activation of quiescent neural stem cells (qNSC) in the aged brain and its consequences in cognitive impairment.** The effects of vitamin D on cognitive decline might be mediated by its capacity to stimulate neurogenesis in the old neurogenic niche. Several factors such as inflammation, and Wnt signaling inhibition facilitate the state of quiescence in NSC diminishing the neurogenic rate [78, 80]. High NSC-Wnt activity leads to longer time in quiescence while enhancing the probability of activation [114]. Vitamin D may activate canonical Wnt signaling through the repression of Wnt inhibitors such as DKK1 and prolonging the time NSC spend in quiescence, increasing their probability to be activated and avoiding being depleted via their differentiation towards astroglial cells [114]. It may be possible that a deficiency in vitamin D results in Wnt signaling imbalance, impairing the gradual activation of NSC required to maintain a neurogenic rate. Thus, hypovitaminosis D might impair these mechanisms leading to a reduction in neurogenesis resulting in cognitive decline.

In conclusion, vitamin D has been shown to exert an important role in neurogenesis and neuronal survival. In hippocampal progenitor cells, vitamin D may potentially act as a co-activator of Wnt/β-catenin pathway to preserve neurogenesis in the aged brain. Thus, the decrease of vitamin D during the senescence processes could have a role in the upregulation of Wnt antagonistic signals responsible of the decrease in neurogenesis that may precede the decline in cognitive performance (summarized in Figure 4), and more studies are required to fully understand the relationship between vitamin D, neurogenesis and cognitive performance in the elderly.
